# Hypogonadism in the HIV-Infected Man

**DOI:** 10.1007/s40506-017-0110-3

**Published:** 2017-02-14

**Authors:** Nicholas Wong, Miles Levy, Iain Stephenson

**Affiliations:** 1 Department of Infectious Diseases and HIV Medicine, Leicester Royal Infirmary, University Hospitals of Leicester NHS Trust, Leicester, LE1 5WW UK; 2 Department of Endocrinology, Leicester Royal Infirmary, University Hospitals of Leicester NHS Trust, Leicester, LE1 5WW UK

**Keywords:** HIV infection, Male hypogonadism, Testosterone deficiency, Androgen deficiency

## Abstract

Low testosterone levels are frequently observed among men with treated and untreated HIV infection. However, the interpretations of biochemical measurements of testicular function are challenging and need to be considered in the context of the clinical presentation and scenario. The distinction between primary and secondary hypogonadism and determination of the underlying clinical pathophysiology are not always straightforward. Early recognition of clinical hypogonadism and appropriate treatment may improve clinical outcomes and quality of life for affected individuals. A principal aim of testosterone replacement is to maintain serum testosterone concentrations in the normal physiological range and should be considered in clinically symptomatic patients.

## Introduction

Hypogonadism commonly affects HIV-positive men and is associated with a variety of clinical symptoms adversely affecting quality of life. However, symptoms of hypogonadism may be non-specific and attributed to a variety of causes, making the diagnosis challenging. An association between HIV infection and hypogonadism has long been recognised, particularly among those with advanced immunosuppression [1–3]. Although the introduction of anti-retroviral therapy (ART) has lowered the incidence of hypogonadism among HIV infected men, it remains an issue, and interpretation of biochemical results and presentation is not straightforward. This article reviews the clinical features and diagnosis of hypogonadism in HIV-infected adult men, illustrated with a series of clinical case descriptions.

## Clinical features of male hypogonadism

Common symptoms of hypogonadism in adult men include fatigue, low mood, reduced libido and erectile dysfunction [4••, 5, 6]. Less frequent complaints include reduced muscle mass, loss of body hair, weight loss, poor sleep, reduced concentration, memory difficulties and increased risk of osteopenia. Physical signs may include loss of axillary and pubic hair, testicular atrophy and gynaecomastia, but these features may be absent among men who develop hypogonadism during adult years, in contrast to pre-pubertal males with hypogonadism where secondary sexual characteristics are undeveloped. Clinical symptoms are not pathognomonic and may indicate other underlying conditions including psychosexual disturbance, diabetes mellitus, neurological disease, chronic hepatitis C virus infection, adverse drug effects (recreational and prescribed), smoking and vitamin D deficiency.

## Pathophysiology

**Fig. 1:**
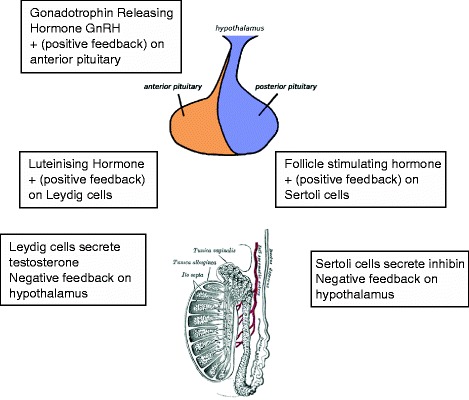
Male hypothalamic-pituitary-gonadal axis

Testosterone is produced by Leydig cells in the testes under stimulation by luteinising hormone (LH) which drives testosterone secretion and follicle-stimulating hormone (FSH), which drives spermatogenesis. Gonadotropins produced by the anterior pituitary gland are regulated by hypothalamus-produced gonadotropin releasing hormone (GnRH). Testosterone inhibits gonadotropin production by direct negative feedback (Fig. 1). Male hypogonadism is the clinical syndrome resulting from the failure of the testes to produce sufficient physiological levels of testosterone (androgen deficiency) and/or reduced numbers of sperm, as a consequence of disruption of the hypothalamic-pituitary-testicular axis. Hypogonadism can be classified into primary hypogonadism as a result of primary testicular disease or secondary hypogonadism as a result of a central defect of the hypothalamus or pituitary gland (Table 1). Distinguishing primary and secondary hypogonadism is readily made by measurement of the serum gonadotropins (LH and FSH). In primary hypogonadism, LH is elevated due to loss of negative feedback, whilst in secondary hypogonadism, LH is inappropriately normal or low.

**Table 1:** Causes of primary and secondary hypogonadism

	Primary hypogonadism	Secondary hypogonadism
Congenital	Klinefelter’s syndrome FSH and LH receptor gene mutations Varicocele Myotonic dystrophy Undescended testicles	Isolated GnRH deficiency (Kallmann syndrome) GnRH deficiency syndrome (genetic disorders with obesity and learning disability)
Acquired	Infection of testis, e.g. mumps Radiation Drugs: ketoconazole, suramin, cytotoxic agents Corticosteroids Testicular trauma or torsion Auto-immune damage to testis Chronic systemic illness including cirrhosis, renal failure, AIDS wasting	Pituitary tumours including adenoma, craniopharyngioma, meningioma, glioma, metastatic deposits Exogenous androgen administration Infiltrative disorder: sarcoid, haemochromatosis Severe acute illness Chronic systemic illnesses Pituitary apoplexy Hyperprolactinaemia Anorexia nervosa

## Biochemical measurements

Regardless of HIV status, the first-line investigation to confirm hypogonadism in adult men is the measurement of early morning serum total testosterone between 8 a.m. and 10 a.m. to account for the diurnal variation of testosterone concentrations which peaks during morning hours [7]. As acute illness may cause transient secondary hypogonadism, men should not be investigated for hypogonadism until the inter-current illness has resolved. Low measurements of total testosterone concentrations are required on at least two separate occasions to confirm hypogonadism.

Serum testosterone circulates in two forms: as protein-bound to sex hormone-binding globulin (SHBG) or as biologically active free testosterone. Routine laboratory assays measure total serum testosterone levels via immunoassay rather than the free hormone; therefore, interpretation must take into account the full clinical and biochemical picture. Measurement of free testosterone is possible via tandem mass spectrometry and is increasingly favoured in academic publications relating to sex steroid measurement, but as it is labour intensive and expensive, it is not currently used in routine clinical practice.

Abnormalities in SHBG can influence the total serum testosterone reading [8], because a low SHBG will lead to a low total testosterone level, even though the free hormone concentration may be normal. Conversely, if the SHBG is elevated, serum total testosterone levels may appear to be within the normal range, despite the fact that free testosterone may actually be low and causing clinical hypogonadism. There are recognised equations that calculate estimated free testosterone concentrations based on albumin, SHBG and total testosterone concentrations which are readily available online [9].

Causes of increased SHBG concentrations include physiological ageing, hypothyroidism, elevated oestrogen levels, chronic liver disease and cirrhosis, HIV infection and drugs that affect liver function. Causes of decreased SHBG concentrations include obesity, insulin resistance and type 2 diabetes mellitus, exogenous androgen, anabolic steroid or glucocorticoid use and nephrotic syndrome. Awareness of the role of SHBG is pertinent to HIV clinicians, as serum SHBG levels are often abnormal in HIV-infected patients [10•]. SHBG may be significantly elevated resulting in normal measurements of total testosterone concentrations, despite low free testosterone levels. Obesity, diabetes and insulin resistance are common reasons for reduced SHBG in HIV patients on treatment, which lowers the serum total testosterone concentration often without affecting free testosterone; weight reduction will correct the binding abnormality.

Once androgen deficiency has been established, measurement of gonadotropin concentrations (LH and FSH) will distinguish primary from secondary hypogonadism. Elevated LH and FSH concentrations indicate primary hypogonadism, i.e. normal hypothalamic and pituitary function with impaired testicular function. Normal or suppressed LH in the presence of testosterone deficiency indicates secondary hypogonadism.

## Primary hypogonadism

Primary hypogonadism is more likely to be associated with gynaecomastia as the stimulatory effects of elevated serum LH and FSH concentrations on the testis increase conversion of testosterone to oestradiol. Primary hypogonadism may be due to congenital abnormalities or acquired disease. Adult HIV clinicians are not likely to diagnosis congenital primary hypogonadism; however, it is worth noting that Klinefelter’s syndrome, characterised by presence of an extra X chromosome (47XXY genotype), is one of the commonest congenital abnormalities to cause primary hypogonadism affecting 1 in 1000 male births [11]. The classical Klinefelter’s phenotype of small testes, increased long bones, psychosocial and learning difficulties is straightforward, but other mosaicisms with lesser numbers of X chromosomes may result in minimal and previously unrecognised clinical features and can present in sexually active men. Diagnosis is made by determination of the karyotype of the peripheral leucocytes.

Acquired primary hypogonadism is often idiopathic with no clear aetiology. HIV-infected patients are at increased risk of any solid organ and haematological malignancies, including those with gonadal involvement [12]. Testicular ultrasound scanning may be needed to identify local abnormalities. Medical oncology treatments including local radiotherapy (e.g. to inguinal nodes for lymphoma) and exposure to alkylating or anti-neoplastic agents can damage testicular tubular cells. Opportunistic infection of the testes is usually clinically apparent and rare with CD4 count >100 cells/mm^3^, but *Mycobacterium tuberculosis*, *Mycobacterium avium*, *Treponema pallidum* and fungal pathogens (*Cryptococcus* spp., *Blastomycosis* spp.) can cause orchitis [13–16]. Reduced testosterone production can follow mumps orchitis, particularly if the infection is bilateral and is contracted in adulthood [17]. Following unwarranted safety concerns of childhood MMR vaccine during the late 1990s, vaccine uptake and coverage rates fell to low levels leading to current re-emergence of mumps infections. HIV infection is known to upregulate tumour necrosis factor and interleukin-1 [18]; both cytokines are linked with decreased testicular steroidogenesis. Autoimmune damage, often in association with thyroid and adrenal insufficiency, may occur driven by cytokine-related inflammation or development of anti-sperm antibodies. Men affected by chronic medical conditions such as liver cirrhosis and end-stage renal disease have an increased incidence of reduced total testosterone concentrations, although suppression of LH and FSH responses in some patients suggests secondary effects may also be contributing [19, 20].

## Secondary hypogonadism

Normal or suppressed gonadotropin concentrations in the presence of low serum testosterone indicate hypothalamic-pituitary dysfunction or secondary hypogonadism; this is the most common cause of hypogonadism among HIV-infected men [6, 21]. 

Disease processes affecting the hypothalamus or pituitary (stalk or gland) can suppress GnRH or gonadotropin secretion, respectively. This may be due to damage to either hypothalamic or pituitary areas from tumours which can be benign (typically causing symptoms by direct compression or by secretion of excess prolactin from pituitary adenomas), primary or metastatic malignant diseases including central nervous system (CNS) lymphoma. Pituitary apoplexy occurs when there is sudden haemorrhage and necrosis into a pre-existing pituitary adenoma, causing pan-hypopituitarism and symptoms associated with mass effect (headache, visual field disturbance). In patients with HIV infection or immunosuppression, lymphoma or syphilis of the pituitary can precipitate or mimic apoplexy [22, 23], and meningeal or pituitary infection with *M. tuberculosis*, *Toxoplasma gondii*, *Pneumocystis jirovecii*, cytomegalovirus (CMV) or candidiasis may result in fibrosis and gradual loss of function [24–26]. Non-infective infiltrating conditions that can cause secondary hypogonadism include sarcoidosis, histiocytosis or iron deposition due to haemochromatosis. The presence of diabetes insipidus may suggest an infiltrative pituitary lesion.

Secretion of gonadotropins may be suppressed not only by specific drugs, such as GnRH antagonists used in prostatic cancer treatment, but also by the use of exogenous androgens (anabolic-androgenic steroids) for recreational body building. HIV-infected men using exogenous androgens to enhance their body image may not admit to their usage, but also be reluctant to withdraw. Drugs, such as stanozolol, suppress gonadotropin secretion and testicular function. Over time, testicular volume may decrease, resulting in impaired infertility. Spermatogenesis, gonadotropin and testosterone secretion remain suppressed for several months after discontinuation, before usually returning to normal [27]. Other medications can suppress gonadotropin secretion, in particular chronic corticosteroid use (e.g. for inflammatory conditions) or opiates including methadone replacement.

Obese patients and those with diabetes or insulin resistance tend to have lower SHBG concentrations and therefore lower serum concentrations of total testosterone [28]. Free testosterone concentrations may be abnormally low, with normal LH and FSH indicating secondary effects. Lifestyle changes and weight reduction generally improve serum testosterone levels. Sex hormone abnormalities appear to play a role in the pathogenesis of insulin resistance and diabetes mellitus in HIV-infected men [29]. Chronic medical conditions including not only HIV infection but also liver cirrhosis, chronic hepatitis C virus infection and renal failure are associated with low testosterone levels and hypogonadism by combination of primary and secondary effects.

## Hypogonadism in HIV-infected men

Hypogonadism is common among HIV-infected men, although the true prevalence remains poorly defined and widely ranging from <10% [30–32] to over 50% [3, 6, 21] in different studies. Before the widespread use of ART, androgen deficiency was the most common endocrine abnormality detected, with low serum testosterone levels in up to 70% of HIV-infected men, with strong associations to low CD4 count (<100 cells/mm^3^), weight loss and AIDS wasting [1–3, 21]. Of these hypogonadal HIV-infected men, around 75% had secondary hypogonadism. As inter-current illnesses suppress gonadotropin secretion, low serum testosterone concentrations in AIDS may reflect active inflammation and disease and overestimate the frequency of hypogonadism. However, studies of symptomatic HIV-infected men, including those receiving ART, also identify low serum testosterone levels in up to 30%, and this is associated with weight loss, muscle wasting with loss of strength and depression [1, 2, 31–33] with levels rising after ART [30]. Conversely, an observational cohort study (CHAMPS) conducted in New York compared testosterone concentrations in 502 HIV-infected and HIV-negative men aged >49 years [6] and found that HIV infection was not associated with low total testosterone levels. However, among HIV-infected men, uncontrolled HIV viraemia (>10,000 copies/ml) was strongly linked to hypogonadism (mainly secondary), with psychotropic and injecting drug use, chronic hepatitis C virus infection and obesity.

Wide-ranging estimates of androgen deficiency are likely to be influenced by SHBG abnormalities in HIV-infected men [29, 31] and available biochemical assays. Free, rather than total testosterone concentration, is a preferred measure, but as previously described, assays are not readily available [10]. Further, low serum testosterone is associated with poor health status in HIV-infected men, with the risk of biochemical hypogonadism cumulatively rising with number of co-morbidities and frailty index scores, raising a suggestion that low testosterone concentrations could be an adaptive change to chronic ill health and reflect ongoing inflammation [33].

Overall, the cause of hypogonadism and low testosterone in HIV-infected men is likely to be multi-factorial related to co-morbidities, chronic inflammation, illicit drug and ART use and body composition changes. Our clinical cases illustrate some of the causes specific to HIV-infected men that may be encountered during clinical practice.

## Case studies

### Case 1: primary hypogonadism

A 57-year-old HIV-infected man who attended for routine HIV clinical review described increased fatigue, reduced libido and erectile dysfunction unresponsive to phosphodiesterase-5 inhibitor use. He had stable type 2 diabetes mellitus (controlled by metformin 1 g b.d). He presented 2 years earlier with pulmonary tuberculosis and HIV-1 infection with nadir CD4 count 50 cells/mm^3^ and viral load >100,000 copies/ml. His HIV infection was well controlled (CD4 540 cells/mm^3^, viral load <40 copies/ml) by Maraviroc 300 mg b.d. and Kivexa (abacavir 600mg/lamivudine 300 mg o.d.). Physical and genital examination was normal. Investigations included early morning total testosterone 6.2 nmol/l (normal range 9.4–37 nmol/l), calculated free testosterone 142 pmol/l (normal range 174–729 pmol/l), LH 16 IU/ml (normal range 1–9 IU/ml), FSH 24 IU/ml (normal range 1–10 IU/ml) and SHBG 22 nmol/l (normal range 15–40 nmol/l). A diagnosis of primary hypogonadism was made. Topical testosterone treatment (Testogel, one sachet per day) was initiated with resolution of symptoms.

### Case 2: primary hypogonadism with drug-induced elevated SHBG

A 54-year-old male, diagnosed with HIV-1 infection 8 years earlier, was well controlled on Atripla (efavirenz 600 mg/emtricitabine 200 mg/tenofovir disoproxil 245 mg) with CD4 count 370 cells/mm^3^ and viral load <40 copies/ml presented with fever, weight loss and shortness of breath. Chest imaging found a large left pleural effusion with left lower lobe consolidation. Fully sensitive *M. tuberculosis* was isolated from pleural specimens. He responded to standard anti-tuberculosis treatment (rifampicin, isoniazid, pyrazinamide and ethambutol). Atripla was continued with additional efavirenz 200 mg o.d. to overcome predicted rifampicin-efavirenz drug interaction. After 2 months, he developed marked reduction in libido with erectile dysfunction. Investigations found serum total testosterone 11 nmol/l, SHBG 170 nmol/l, calculated free testosterone 60 pmol/l, LH 14 IU/ml and FSH 15 IU/ml. Efavirenz has a direct oestrogenic effect by modulating oestrogen receptors [34], and this effect may have been exacerbated by SHBG elevation (HIV infection, anti-tuberculosis treatment and ART) reducing free testosterone concentrations, despite normal total levels. After additional efavirenz was discontinued, SHBG returned to normal and his clinical symptoms fully resolved.

### Case 3: opportunistic HIV-related tuberculous CNS infection leading to pituitary dysfunction

**Fig. 2:**
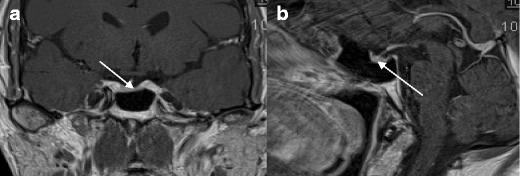
Case 3 (pituitary fibrosis secondary to tuberculous CNS infection). **a** Coronal MRI showing low volume pituitary gland. **b** Sagittal MRI showing low volume pituitary gland and probable pituitary fibrosis

A 51-year-old male with stable HIV infection (current CD4 350 cells/mm^3^, viral load <40 copies/ml) managed on Triumeq (dolutegravir 50 mg/abacavir 600 mg/lamivudine 300 mg) had been diagnosed 9 years earlier when presenting with disseminated tuberculosis (sites of involvement: pulmonary, renal, meningeal). He also had sero-positive rheumatoid arthritis. He described ongoing lethargy, fatigue and loss of muscle strength. Serum total testosterone was 2.1 nmol/l, calculated free testosterone 46.1 pmol/l, LH 5 IU/l, FSH 10 IU/l, SHBG 21 nmol/l, thyroxine 13 pmol/l (normal range 9–25 pmol/l), cortisol 394 nmol/l (normal range 138–620 nmol/l) and prolactin 74 mIU/l (normal range 50–400 mIU/l). Magnetic resonance imaging (MRI) of the pituitary found a low volume pituitary gland with hypointense parenchyma consistent with healed granulomatous lesion (Fig. 2). Symptoms resolved following intramuscular testosterone replacement.

### Case 4: pituitary haemorrhage from direct CMV infection

**Fig. 3:**
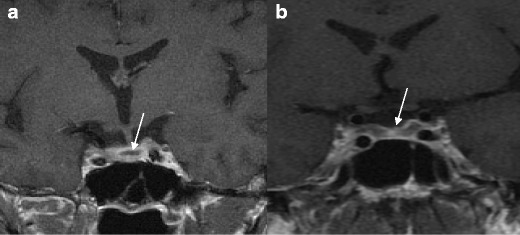
Case 4 (suspected HIV-related pituitary haemorrhage). **a** Coronal MRI at presentation showing likely haemorrhage within enlarged pituitary gland. **b** Six-month follow-up MRI showing reduction in pituitary size with concave upper border

A 49-year-old man presented with sudden confusion and headache. He was diagnosed HIV-positive (CD4 220 cells/mm^3^, viral load 3898 copies/ml) with acute kidney injury and CMV viraemia. Computerised tomography (CT) brain imaging was normal. He received once daily lamivudine 300 mg, darunavir 800 mg and ritonavir 100 mg with raltegravir 400 mg b.d. He developed symptomatic hypotension. Testosterone was <0.3 pmol/l with biochemical features of panhypopituitarism with cortisol <25 nmol/l, free thyroxine 4.6 pmol/l, TSH <0.05 IU/l, prolactin <10 mIU/l, LH 1.2 IU/l and FSH <0.5 IU/l. Cerebrospinal fluid (CSF) analysis revealed a raised protein (0.66 g/l) and negative microscopy. MR imaging found evidence of subacute haemorrhage in the pituitary gland, with no evidence of pituitary tumour, suggesting direct haemorrhage into the gland, which is unusual (Fig. 3). The working diagnosis was CMV infection of the pituitary gland causing haemorrhage [26]. His hypopituitarism was treated by appropriate endocrine replacement with hydrocortisone, levothyroxine and testosterone.

### Case 5: exogenous anabolic steroid use

A 49-year-old heterosexual man who was diagnosed with HIV infection 3 years earlier following screening when hospitalised for pneumococcal pneumonia was managed with once daily darunavir 800 mg, ritonavir 100 mg and emtricitabine 200 mg/tenofovir disoproxil 245 mg. His current CD4 level was 370 cells/mm^3^ and viral load was <40 copies/ml. He trained regularly at the gym and was a keen amateur boxer. He wished to conceive with his wife but complained of lack of energy and reduced libido. Serum total testosterone levels were 5.5 pmol/l, calculated free testosterone 150 pmol/l, SHBG 14 nmol/l, FSH 4 IU/ml, LH 5 IU/ml and prolactin 89 mIU/ml. He admitted to regular use of oxandrolone and stanozolol over several years.

## Discussion of cases

These cases represent a range of real-life clinical scenarios whereby patients have low testosterone concentrations and HIV infection. Primary hypogonadism, as illustrated by case 1, is readily recognised by the presence of elevated LH levels which makes the decision to treat with testosterone straightforward, as it is clear that the pituitary is trying to compensate for the low testosterone. Whilst it is not possible to identify whether his HIV infection or an alternative pathology caused the primary hypogonadism, the patient clinically responded to testosterone replacement. Case 2 is an important learning point as efavirenz appeared to cause reversible symptoms of hypogonadism. The hypothesis is that efavirenz-induced oestrogen antagonism, in addition to a drug-induced elevation of SHBG reducing circulating free testosterone levels. Dose reduction improved both SHBG abnormalities and resolved the clinical symptoms. This shows the importance of interpreting serum testosterone levels in the context of drug therapy and clinical scenario. Cases 3 and 4 illustrate the potential for HIV-associated opportunistic infections to directly cause hypothalamic-pituitary pathology. In case 3, it is likely that CNS tuberculosis caused pituitary disease leading to a shrunken fibrotic gland (Fig. 2). The unusual MRI appearance in case 4 of a haemorrhagic pituitary gland in the absence of a pituitary tumour suggests direct CMV infection of the gland, which has been previously described [26] (Fig. 3). Case 5 illustrates how exogenous androgenic steroid use suppresses testosterone concentrations, but may not be volunteered by the patient unless specifically questioned.

## Clinical management

**Fig. 4:**
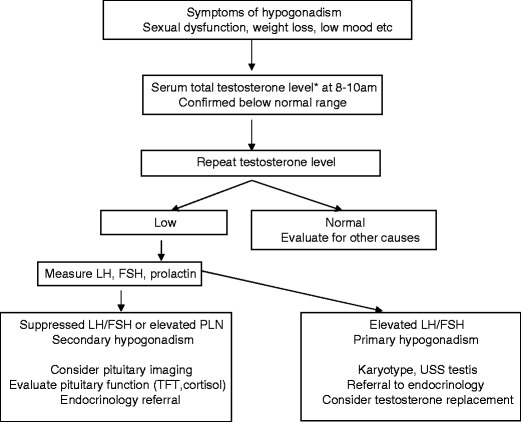
Evaluation of patient with hypogonadism. *Asterisk* indicates free testosterone if available

Once hypogonadism has been confirmed and evaluated (Fig. 4), appropriate testosterone replacement should be considered. The aims of treatment are to improve clinical symptoms, reduce the risk of osteoporosis and maintain serum testosterone concentrations in the normal healthy male reference range. The main long-term consequence of untreated true hypogonadism is reduced bone density and osteoporosis, which may be compounded by the use of tenofovir disoproxil which is also associated with bone demineralisation [35]. Dual-energy X-ray absorptiometry (DEXA) scanning to assess bone density is recommended in HIV-positive men over 70 years of age or in those over 50 years who have additional risk factors such as previous bone fracture, chronic hepatitis virus co-infection or hypogonadism [36].

Systematic review of randomised, double-blinded placebo-controlled studies assessing testosterone replacement among androgen-deficient HIV-infected men with weight loss has found significant increases in measured fat-free mass, lean body mass and muscle mass [37•]. Fifty-one HIV-infected men (mean age 42 years) with wasting were randomised to intramuscular testosterone or placebo for 6 months [38]. Those men receiving androgen therapy had significant increases of 1.9, 2 and 2.4 kg in lean body mass, fat-free mass and muscle mass, respectively. Transdermal testosterone replacement, for 3 months, among 41 HIV-infected men aged 18–60 years with weight loss increased lean body mass by 1.35 kg as compared to placebo [39]. The clinical significance of small gains in lean body mass is unclear, although secondary endpoints such as muscle strength, fatigue, mood and health-related quality of life scores are generally improved [39–42]. Furthermore, whilst well tolerated with no observed adverse effects on CD4 or HIV viral load control, studies of testosterone replacement in HIV-infected men have been of relatively short duration (3–6 months), so long-term benefits and potential adverse effects of therapy are unclear. Current Endocrine Society clinical practice guidelines suggest at least short-term testosterone therapy as adjunctive therapy in HIV-infected men with weight loss and low testosterone levels in order to promote gain in muscle strength and lean body mass [4••].

Testosterone replacement may cause growth in androgen-sensitive malignancies such as prostatic or breast and is infrequently associated with polycythaemia. Monitoring of serum haematocrit and prostate gland via digital rectal examination and serum prostate-specific antigen levels is recommended whilst on testosterone replacement. Other adverse reactions include local skin reactions from topical therapy or injection site reactions.

In summary, symptoms of hypogonadism and low testosterone levels are common in HIV-infected men and are frequently overlooked. Interpretation of biochemical testing is challenging. Once confirmed, causes of hypogonadism should be investigated and appropriate treatment may be needed.
